# Preclinical Assessment of a New Virus-like Particle-Based Quadrivalent Human Papillomavirus Vaccine in Animal Models

**DOI:** 10.3390/vaccines14010066

**Published:** 2026-01-05

**Authors:** Hajar Mohammadi Barzelighi, Zahra Naderi Saffar, Erfan Pakatchian, Mohammad Taqavian, Babak Javadimehr, Mansooreh Safaeian, Payam Abbaszadeh, Hasan Jalili

**Affiliations:** 1 Research and Development Department, Biosunpharmed Company, Tehran 1436935359, Iran; ha.mohammadi@biosunpharmed.com (H.M.B.); payam.vet.med@gmail.com (P.A.); 2 Department of Life Sciences, Faculty of New Sciences and Technologies, University of Tehran, Tehran 1417935840, Iran

**Keywords:** HPV, immunogenicity, single-dose and repeated-dose toxicity, reproductive toxicity, vaccine safety

## Abstract

**Background:** A quadrivalent HPV vaccine (BPV) has been developed to prevent diseases caused by HPV types 6, 11, 16, and 18 for the first time in Iran. The BPV is composed of the papillomavirus major capsid protein L1, which serves as the primary target in the design of the prophylactic HPV vaccines. To enhance immunogenicity, BPV was formulated with an amorphous aluminum hydroxy phosphate sulfate adjuvant. **Methods:** The immunogenicity and safety of BPV were assessed through analyses of both humoral and cell-mediated immunity, single and repeated doses, and reproductive effects using animal models. **Results:** Acute toxicity assessments showed no abnormalities in ophthalmic examinations, biochemical profiles, hematological parameters, and gross pathology findings. Additionally, no mortality or abnormal clinical signs were observed during a 90-day repeated-dose toxicity study. While some inflammatory reactions were noted at the injection sites and in the liver tissues of BPV-treated groups, these reactions were resolved by day 90 after the initial BPV administration. Furthermore, no signs of toxicity were detected in F1 offspring, and no adverse effects were identified in maternal reproductive performance, fertility, or hematological or biochemical parameters throughout the study duration. The BPV candidate successfully induced T-cell proliferation and increased the proportions of CD_3_^+^ CD_4_^+^ and CD_3_^+^ CD_8_^+^ T cells. It also stimulated the secretion of both interferon gamma (IFN-γ) and interleukin-4 (IL-4) cytokines in splenocytes isolated from animal models after the third dose. Moreover, anti-HPV L1 IgG antibody production was confirmed on day 14 after administration of each of the three BPV vaccine doses. **Conclusions:** The findings suggest that BPV is a vaccine candidate that stimulates both cellular and humoral immunity and demonstrate its safety profile in animal models.

## 1. Introduction

Human papillomavirus (HPV) is a viral infection of the female reproductive tract and a major cause of sexually transmitted infections. This small, non-enveloped DNA virus typically infects epithelial cells [1]. The development of cervical cancer is strongly associated with HPV infections, with the virus implicated in 99.7% of the reported cases [2]. Cervical cancer is the second most predominant type of cancer among women around the world, particularly in developing countries, where it poses a significant public health concern [3].

HPV is composed of a group of viruses, of which more than 200 are closely related and more than 40 are transmitted through direct sexual contact [4]. Low-risk or non-oncogenic HPV types—6, 11, 42, 43, and 44—can cause benign or mild cervical cell changes, anogenital warts, and respiratory tract papillomas [4]. Over 90% of anogenital wart cases are attributed to low-risk HPV 6 or 11 [1,4]. High-risk or oncogenic HPV types—16, 18, 31, 33, 34, 35, 39, 45, 51, 52, 56, 58, 59, 66, 68, and 70—are strongly correlated with cervical and other anogenital cancers. These HPV types are found in 99% of cervical precancerous abnormalities [1,5]. A 2021 report from the Centers for Disease Control and Prevention [6] indicated that HPV 16 and 18 are responsible for about 80% of cervical cancer cases [7], and HPV 16 alone accounted for nearly 50% of global cervical cancer. Moreover, five other high-risk HPV types—31, 33, 45, 52, and 58—contribute to another 15% of cervical cancers and 11% of all HPV-related cancers [1,6].

Currently, there are six licensed HPV vaccines in the market all over the world: three bivalent vaccines, two quadrivalent vaccines, and one nonvalent vaccine. All these vaccines provide protection against high-risk HPV 16 and 18, and their safety and effectiveness in preventing HPV infection and cervical cancer have been demonstrated [6,8]. Commercial HPV vaccines, particularly Gardasil, Gaedasil9, Cervarix, Cecolin, Walvax, and Cervavac, are subunit vaccines comprising HPV virus-like particles (VLPs) [9,10,11]. These particles are formed from the papillomavirus major capsid protein L1, which has a molecular weight of approximately 55 kDa and is capable of self-assembling into immunogenic structures that induce neutralizing antibodies. Vaccine candidates developed via recombinant DNA technology produce VLPs that closely resemble the natural structure of HPV [9,10,11]. These vaccines utilize the HPV L1 antigen to trigger a protective immune response without the risk of infection [12,13,14].

In 2023, a quadrivalent HPV vaccine, (BPV), was manufactured for the first time in Iran, following the importation of 348,305 vials of Gardasil and the sale of 116,603 vials of the Iranian dual-dose vaccine. This initiative was driven by high demand for vaccination due to the widespread prevalence of the virus. The BPV contains HPV L1 types 6, 11, 16 and 18 [15,16]. The distribution of HPV genotypes in Iran mirrors global patterns, with types 6 and 16 being the most prevalent among low-risk and high-risk genotypes, respectively.

Regulatory non-clinical testing is essential for transitioning a vaccine candidate from laboratory research to clinical trials. In this light, preclinical studies were conducted in mice and rats to evaluate the safety, toxicity, and immunogenicity of the BPV candidate relative to Gardasil [17]. This study was designed to assess the capacity of the BPV to stimulate immune responses by measuring its effects on T-helper and T-cytotoxic lymphocyte proliferation, Th1-type interferon gamma (IFN-γ), Th2-type interleukin-4 (IL-4), and specific antibody titers in BALB/c mice. In addition, acute and chronic toxicity, reproductive toxicity, and local tolerance were evaluated in BALB/c mice and Wistar rats. Acute toxicity testing examines short-term adverse effects after vaccine administration, while chronic toxicity testing evaluates long-term safety, including delayed reactions, cumulative toxicity, and reproductive impacts. Together, these studies establish a complete safety profile beyond the initial immune response. By assessing both immediate and prolonged outcomes in animal models, researchers reduce risks before human trials, protect clinical participants, and build public trust. For HPV vaccines, such rigorous testing is essential to ensure their cancer-preventive benefits are not compromised by unexpected risks [17].

## 2. Materials and Methods

### 2.1. Animal Maintenance and Housing

This study was adhered to the guidelines established by the Institutional Animal Care and Use Committee of the Pasteur Institute in Iran to ensure the ethical treatment and welfare of the animals involved [18]. Throughout this study, the health and behavior of the animals were closely monitored, particularly after injections, to detect any adverse reactions. This strategy allowed for the assessment of both the efficacy and potential side effects associated with the BPV and control treatments [18].

### 2.2. Animal Grouping and Treatment

The control and test substances used in this study were as follows: BPV (group A), Gardasil quadrivalent vaccine (GV; as the positive control, group B), phosphate-buffered saline (PBS, 1×; as the negative control, group C), and BP aluminum adjuvant (BAA; group D). All formulations were stored at 2–8 °C and administered via an insulin syringe into the right flank of the animals. Each 0.5 mL dose of BPV contained VLPs at concentrations of 20 µg/mL for HPV types 6 and 18 and 40 µg/mL for types 11 and 16. The BPV formulation (group A) also included sodium chloride (0.33 M), L-histidine (0.01 M), Polysorbate 80 (0.08 M), sodium tetraborate decahydrate (0.18 M), and BAA (with an aluminum content of 240 µg/mL). Group B received the GV formulation (Merck, Kenilworth, NJ, USA), while group D received BAA alone at the same aluminum concentration. The PBS control (group C) consisted of 137 mM of NaCl, 2.7 mM of KCl, 10 mM of Na_2_HPO_4_, and 1.8 mM of KH_2_PO_4_. Antigenic content for each group was analyzed separately (Table 1). This study entailed four evaluation categories: immunological assays (70 days), single- and repeated-dose toxicity (70–90 days), reproductive toxicity (90 days), and local tolerance (60 days) (Table 1). Efficacy and repeated-dose toxicity assessments were conducted on female BALB/C mice aged 6–10 weeks and weighing 20–22 g. Reproductive toxicity and local tolerance evaluations were carried out on female Wistar rats with an average age of eight weeks. All animals were closely observed for changes in behavior, appearance, food consumption, and stool shape throughout the study.

**Table 1:** Overview of experimental procedures and immunization schedule.

Study Design			Animal	Content of Injection Groups				Injection Schedule (Days)
				BPV **	GV	PBS	BAA	
Immunological assays	Cellular *	72	BALB/c mice	A_1_: 1:5 of human dose: 24 µg L1 (4, 8, 8, and 4 µg for HPV 6, 11, 16, and 18, respectively), BAA with Al content 48 µg	B_1_: 1:5 of human dose	C_1_: 1× PBS buffer	D_1_: Al content equal to 48 µg	0, 14, and 56
	Humoral	72						0, 14, and 56
Single-dose toxicity		72	BALB/c mice	A2: 56 µg L1 (16, 16, 8, and 16 µg for HPV 6, 11, 16, and 18, respectively), BAA with Al content 112 µg	B2: 56 µg of L1 via dilution of GV	C1: 1× PBS buffer	D1: Al content equal to 112 µg	0
Repeated-dose toxicity		72						0, 14, and 56
Reproductive toxicity		24	Wistar rats	A_3_: 120 µg L1 (20, 40, 40, and 20 µg for HPV 6, 11, 16, and 18, respectively), BAA with Al content 240 µg	B_3_: 120 µg of L1	C_3_: 1× PBS buffer	D_3_: Al content equal to 240 µg	35 and 14 days before cohabitation, GD_6_ and LD_7_
Local tolerance		24	Wistar rats					0, 14, and 56

**Figure 1:**
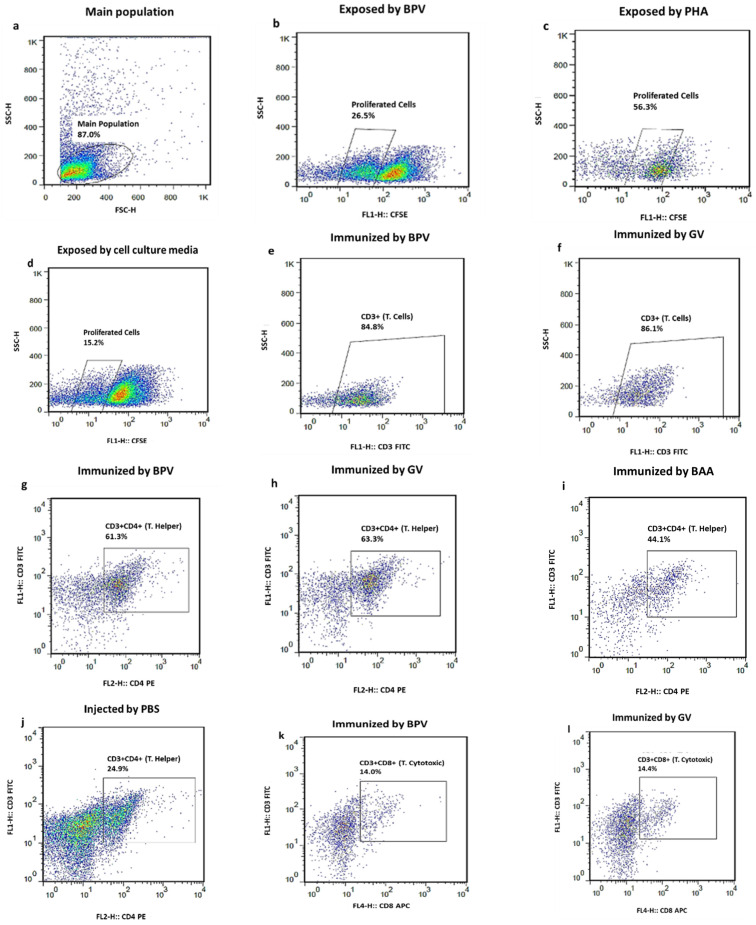

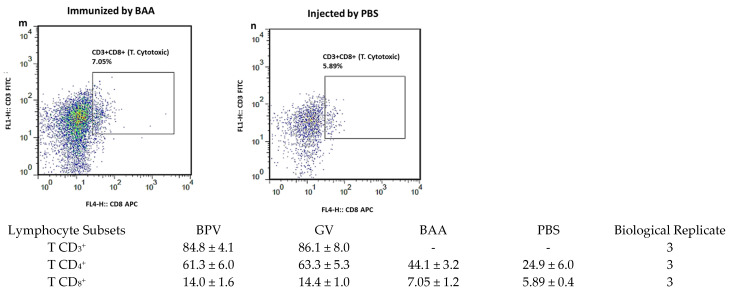
In vitro lymphocyte proliferation assay in BALB/c mice treated with various formulations (BPV, GV, PBS, and BAA). (**a**–**d**) Representative wells showing splenocytes treated with BPV, PHA (positive control), and cell culture medium (negative control). (**e**,**f**) Expansion of CD3^+^ T lymphocytes following BPV treatment. (**g**–**j**) Proliferation of CD4^+^ T helper cells, and (**k**–**n**) proliferation of CD8^+^ cytotoxic T cells, assessed by CFSE dilution. Splenocytes were stained with FITC-conjugated anti-mouse CD3, PE-conjugated anti-mouse CD4, and APC-conjugated anti-mouse CD8a antibodies. Distribution and proliferation of lymphocyte subsets (**e**–**n**). Data are presented as mean ± SEM (n = 3) and compiled in the table beneath the figure. Proliferating cells were identified as CFSE^+^ within each subset. Appropriate single-stained compensation controls and FMO controls were included to ensure accurate gating.

**Figure 2:**
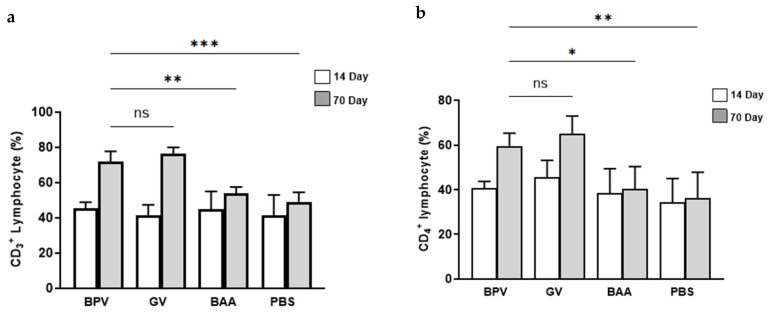

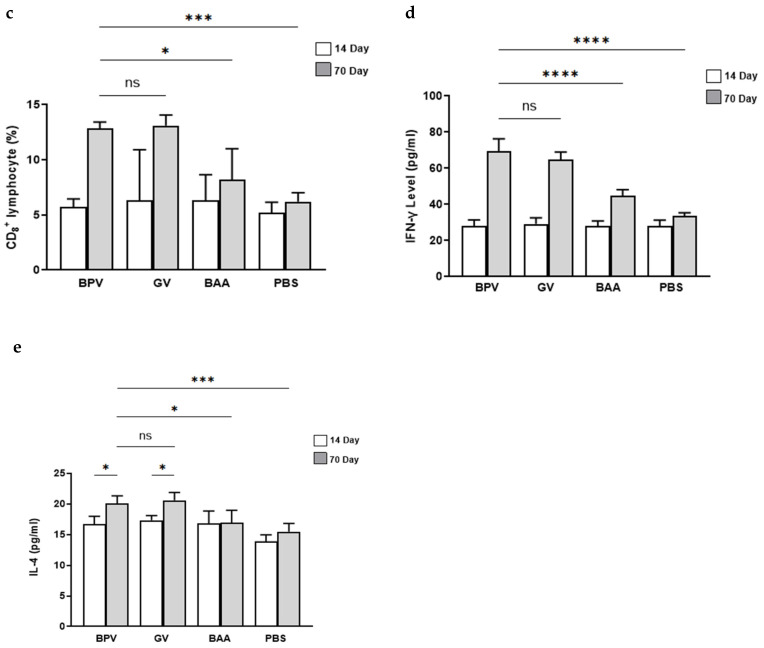
Percentages of lymphocyte subsets—(**a**) CD3^+^, (**b**) CD4^+^, and (**c**) CD8^+^—and cytokine levels—(**d**) IFN-γ and IL-4 (**e**)—in splenocytes following BPV stimulation on days 14 (white bars) and 70 (gray bars). Cellular immune response data were assessed for normality using the Shapiro–Wilk test and, when normally distributed, analyzed using one-way ANOVA followed by Tukey’s post hoc test. Each group consisted of five animals (n = 5). Data are presented as mean ± SD. * *p* < 0.05; ** *p* < 0.01; *** *p* < 0.001; **** *p* < 0.0001; ns, not significant.

* Splenocytes of BALB/c mice immunized with BPV (group A_1_), GV as a positive control (group B_1_), PBS as a negative control (group C_1_), or BAA (D_1_) were used for lymphocyte proliferation assay and ELISA (Figure 1 and Figure 2). ** The injection formulations for each group were prepared in an additional buffer composed of sodium chloride (0.33 M), L-histidine (0.01 M), polysorbate 80 (0.08 M), and sodium tetraborate decahydrate (0.18 M).

### 2.3. Toxicology

All clinical signs and behavioral changes in animals were recorded twice daily, with any abnormality documented at their onset and throughout the study. Dermal reactions at the injection site were assessed on scheduled injection days (Table 1). Erythema, eschar, and edema were observed and graded as follows: no erythema and edema and very slight, moderate, and severe erythema and edema (eschar formation) [19]. Body weights and food consumption of the animals were recorded both before the initiation of treatment and throughout this study for all groups. Blood samples were collected for further investigation.

#### 2.3.1. Evaluation of Single-Dose (Acute) Toxicity

The single-dose toxicity study involved 72 BALB/c mice, which were categorized into four groups: A_2_ (BPV), B_2_ (GV), C_2_ (PBS), and D_2_ (BAA) (Table 1). Each group consisted of 18 mice, equally divided between males and females. Six mice per group received an injection of 56 μg of total protein, approximately 1200× the projected human dose per kg body weight for the vaccine-treated groups. Another six mice received sterile PBS, while an additional six mice were given BAA containing 112 μg of aluminum at three time points (days 0, 14, and 56) [20]. Morbidity and mortality were evaluated at 1, 2, 3, 6, 12, and 24 h after vaccine exposure. Additionally, the body weights of the mice were monitored for two weeks. Following this period, the mice were euthanized, and specimens were collected for hematological and biochemical analyses, including measurements of alanine aminotransferase, aspartate aminotransferase, alkaline phosphatase, urea, and creatinine [17,19]. For gross pathology evaluation, the internal organs—including the spleen, genital organs, heart, muscle, brain, kidneys, liver, and lungs—were weighed after examining the external surface of the body. In the toxicological experiments, the organ weights from BPV-treated mice were compared to those from the PBS control group, which served as the negative control [19].

#### 2.3.2. Evaluation of Repeated-Dose (Sub-Chronic) Toxicity

For the repeated-dose toxicity study, 72 male and female BALB/C mice were administered the same concentrations as in groups A_2_–D_2_ via an intramuscular injection on days 0, 14, and 56 (Table 1). The mice were divided into four groups of 18 animals each. At each time point, the groups received individually the following doses: 56 µg of BPV (group A_2_), GV (group B_2_), PBS (group C_2_), and BAA (group D_2_). The evaluations were based on the effects of repeated exposure to these three doses. For biochemical and hematological analyses, blood samples were collected on days 35, 60, and 90. Ophthalmic toxicity was assessed via indirect ophthalmoscopy before the first injection and again before necroscopy on days 35 and 60 [19]. Internal organs—including the spleen, genital organs, heart, muscle, brain, kidney, liver, and lungs—were isolated and examined for gross pathology and histopathology. For histopathological analysis, five mice were randomly selected from each group. Their organs were weighed and fixed in a 4% formaldehyde solution. Microscopic examinations were conducted on hematoxylin and eosin-stained paraffin-embedded tissue sections [19]. Histopathological examination continued through day 90.

#### 2.3.3. Reproductive Toxicology Analysis

Reproductive toxicity was assessed over 90 days using 24 female Wistar rats [17]. The animals received intramuscular injections of BPV and GV at a dose of 120 µg (300 times the projected human dose) [21,22]. Control groups were given PBS (group C_3_) or BAA containing 240 µg of aluminum (group D_3_) five and two weeks before cohabitation on gestation day 6 and lactation day 7 (Table 1) [17]. For mating, two female rats and one healthy male rat were placed in separate cages for three consecutive days and nights. On day 35 of this study, pregnant females gave birth between 17 and 22 days after mating. Injections were administered based on the planned schedule. The effects of each treatment group were evaluated according to several criteria, including mating performance, appearance, mobility, food consumption, stool shape, physical damage, and fetal body weight [17]. On day 90, mothers from each group and three offspring from each mother were randomly selected for blood collection via cardiac puncture to assess biochemical and hematological factors [17,23]. On the same day, all animals underwent a comprehensive pathological examination.

### 2.4. Assessment of Local Tolerance in Rats

Skin reactions at the injection site were monitored in 24 Wistar rats, divided into four groups (vaccine-treated, BAA-treated, and PBS control) over a 90-day period. The assessment followed a repeated-dose toxicity protocol using doses that were 300 times higher than the human antigen dose (120 µg for BPV and GV and 240 µg for the aluminum content in BAA). Injections were administered intramuscularly on days 0, 14, and 56 (Table 1) [17,23], and the signs of redness, swelling, and wounds were examined at intervals ranging from 1 h to 48 h post-injection.

### 2.5. Evaluation of BPV Efficacy in BALB/c Mice After Immunization

Immunogenicity and immune response were assessed in 72 BALB/c mice over 70 days. The animals were randomly divided into four groups (18 mice each): A_1_, B_1_, C_1_, and D_1_, which received BPV, GV, PBS, and BAA, respectively (Table 1). All the mice groups received three intramuscular injections of antigenic doses equivalent to one-fifth of the human dose on days 0, 14, and 56 [17].

#### 2.5.1. Assessment of Spleen Lymphocyte Proliferation and Cytokine Production

Female BALB/c mice were humanely killed via intraperitoneal injection of thiopental sodium on days 14 and 70 following intramuscular immunization (Table 1). Splenocytes were extracted using a mechanical technique, and erythrocytes were lysed with a lysis buffer (Cytomatin gene, Tehran, Iran) according to the manufacturer’s instructions [17]. Isolated cells were seeded into 96-well culture plates at a density of 2 × 10^5^ cells per well in RPMI-1640 medium (Gibco, New York, NY, USA) supplemented with 10% fetal bovine serum (FBS; Gibco, New York, NY, USA). All experiments were conducted in triplicate. Cell culture medium was used as the negative control, while 1 µg/mL of phytohemagglutinin (PHA; Gibco, New York, NY, USA) was used as the positive control. For the stimulation test, 15 µg/mL of BPV was employed to stimulate splenocytes [24]. Cell proliferation was assessed by labeling the cells with carboxyfluorescein succinimidyl ester (Biolegend, San Diego, CA, USA) [17]. Cell suspensions were kept in Dulbecco’s phosphate-buffered saline (DPBS) containing 2% FBS and washed three times with DPBS [24]. The cells were then centrifuged at 3000 rpm for 10 min. Afterwards, the percentages of CD3^+^, CD4^+^, and CD8^+^ lymphocytes were determined using a BD FACS Calibur flow cytometer (BD Biosciences, East Rutherford, NJ, USA). Antibodies used for the flow cytometry analysis included PE anti-mouse CD4, FITC anti-mouse CD3, and APC anti-mouse CD8a (all from BioLegend, San Diego, CA, USA). Culture supernatants were collected and stored at −20 °C for the evaluation of IFN-γ and IL-4 levels using a sandwich ELISA, following the manufacturer’s instructions [25]. Lymphocyte populations were gated using FSC-H vs. SSC-H to exclude debris and isolate singlets. Proliferation was assessed with CFSE labeling, where dividing cells showed reduced fluorescence. T cell subsets were identified by CD3, CD4, and CD8 surface markers. Single-stained and FMO controls were applied to correct spectral overlap and define gating thresholds. Data on total T cells, CD4+, CD8+, and proliferating cells were analyzed in FlowJo vX and reported as mean ± SEM, based on at least three biological replicates per group.

#### 2.5.2. Serological Analysis

The humoral immune response was evaluated in 72 BALB/c mice using one-fifth of the human vaccine dose (24 µg total L1 protein per injection). The mice were divided into different groups: A_1_ (BPV), B_1_ (GV, positive control), C_1_ (PBS, negative control), and D_1_ (BAA) (Table 1). Each group received three intramuscular injections into the right leg on days 0, 14, and 56 (Table 1) [17]. Blood samples of immunized mice in all groups were collected from the retro-orbital plexus under anesthesia for serum separation. To assess immunogenicity, indirect ELISA was performed for all groups using an HPV kit (Alpha Diagnostic, San Antonio, TX, USA), following the manufacturer’s instructions. Anti-L1 antibody levels against HPV serotypes 6, 11, 16, and 18 were quantified on days 35 and 70 after the initial injection. According to the kit protocol, a sample was considered positive if its net optical density ratio to that of the calibrator exceeded 1 U/mL.

### 2.6. Statistical Analysis

Statistical analyses of data were performed using GraphPad Prism (version 9) to assess the immunogenicity and toxicological effects of BPV. All datasets were first examined for normality using the Shapiro–Wilk test, with *p*-values greater than 0.05 considered indicative of a normal distribution. For data meeting the assumption of normality, parametric tests including Student’s t-test and one-way analysis of variance (ANOVA) were applied. In cases where the data did not conform to a normal distribution, non-parametric methods were employed, specifically the Kruskal–Wallis test, to evaluate differences among groups. A *p*-value of 0.05 was considered statistically significant.

## 3. Results

The major capsid protein L1 of HPV is a ~55 kDa protein that self-assembles into VLPs. L1 can stimulate the production of specific antibodies in vaccinated hosts. These VLPs closely resemble the natural structure of HPV, making L1 protein the primary target in the development of prophylactic HPV vaccines. In this study, we examined BPV, a new quadrivalent VLP-based vaccine composed of L1 from HPV types 6, 11, 16, and 18. To enhance its immunogenicity, we formulated the vaccine with an amorphous aluminum hydroxy phosphate sulfate adjuvant. Throughout this study, no changes were observed in the behavior, appearance, food consumption, stool shape, or body weight of the BALB/c mice.

### 3.1. Toxicology

#### 3.1.1. Evaluation of Single-Dose (Acute) Toxicity

This assessment focused on potential mortality and unusual clinical symptoms following a single intramuscular administration of BPV over a 14-day period. The results showed that a single dose of BPV did not affect the survival rates of animals. No treatment-related changes were observed in physical appearance, clinical signs, mobility, stool shape, physical damage, body weight, food consumption, or ophthalmic examinations in either the BPV or BAA group (Table 1). These findings were consistent with those observed in the BPV and GV groups. No macroscopic abnormalities were detected in the organs or tissues of mice treated with BPV or the BAA alone. Across all assessments, we observed no significant differences in biochemical, hematological, or gross pathology parameters between the BPV and GV groups.

#### 3.1.2. Evaluation of Repeated-Dose (Sub-Chronic) Toxicity

Overall toxicity and local reactions were assessed in four (BPV-treated, GV-treated, BAA-treated, and PBS control) groups. No significant changes related to BPV and BAA were noted in terms of appearance, clinical signs, mobility, stool shape, physical damage, body weight, food consumption, ophthalmic examinations, or injection site reactions (such as swelling and redness). Additionally, no mortality occurred throughout the study and after the final injection, resulting in a 0% mortality rate. Due to several biochemical and hematological parameters exhibiting considerable variability (reflected by large standard deviations), the assumption of normality was not satisfied. Consequently, comparisons across the four experimental groups (BPV, GV, PBS, and BAA) were conducted using the non-parametric Kruskal–Wallis test. Results are expressed as mean ± SD, with statistical significance defined at *p* < 0.05. Male samples were not included in the final analysis regarding to sample lost.

**Figure 3:**
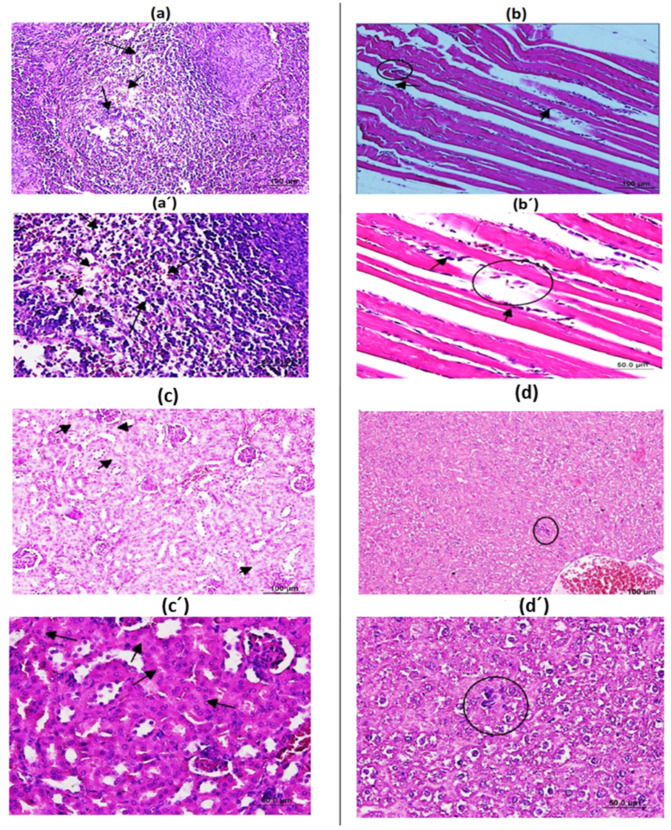
Histopathological evaluation of spleen, muscle, kidney, and liver following three administrations. (**a**,**a′**) Moderate lymphoid depletion observed in the splenic white pulp (black arrows). (**b**,**b′**) Mononuclear cell infiltration accompanied by invasion of muscle fibers (circle). (**c**,**c′**) Cellular swelling within the renal tubular epithelium (black arrows). (**d**,**d′**) Focal inflammatory changes in the liver parenchyma with infiltrating inflammatory cells (circle). In all figures, magnification is shown at 100 µm in the upper row and 50 µm in the lower row. N: 6 mice for each group.

Statistically significant changes (*p* < 0.05) were detected in biochemical markers (ALT, AST, ALP, GLU, BUN, Crea, and urea) and hematological parameters (RBC, Hgb, Hct, MCV, MCH, MCHC, PLT, and differential leukocyte counts including neutrophils, monocytes, and eosinophils) in the BPV and BAA groups compared to the PBS group on days 60 and 90 (*p* < 0.05; Table 2). Moderate increases in leukocytes, neutrophils, monocytes, and globulin were observed in both male and female mice treated with BPV and BAA on day 60, which normalized by day 90. There were no BPV-related changes in the other hematological or biochemical parameters. The results for mice immunized with BPV and GV were similar (*p* > 0.05). At final necropsy, statistically significant increases in liver and spleen weights were found in both male and female mice treated with BPV or the BAA. While spleen weights were within normal limits at the recovery necropsy after the 90-day period, liver weight differences in BPV compared to PBS controls remained within the normal variation range and were not linked to treatment. Histopathological analysis on day 35 revealed slight lymphoid depletion in the white pulp of the spleen, swelling of tubular epithelial cells, and the presence of macrophages and granulocytes containing granular material, along with light necrotic skeletal myofibers at the injection site. The mild infiltration of inflammatory cells into the liver parenchyma was observed in 4 (80%), 1 (20%), 5 (100%), and 3 (60%) of the BPV-treated mice, respectively (Figure 3). By day 60, lymphoid depletion and epithelial swelling had recovered, and by day 90, both injection site inflammation and liver infiltration had recovered. The BAA-treated groups exhibited similar findings to those observed in the BPV-treated group.

#### 3.1.3. Reproductive Toxicology Analysis

Mating performance, growth, fertility, and fetal survival were evaluated in female Wistar rats over a 90-day period. No significant changes were observed in mating behavior, appearance, mobility, food consumption, stool shape, physical damage, fertility rates, fetal survival, or maternal and neonatal mortality in the BPV and BAA-treated groups. Maternal weight gain was found to correlate with fetal weight and food intake.

The gross examination of maternal thoracic and abdominal organs revealed minor, non-adverse findings with no dose dependency and no pathological significance in the BPV- and BAA-treated groups. These findings were within the normal biological variation for Wistar rats, were not corroborated by histopathological examination, and had no impact on reproductive or developmental endpoints; furthermore, there was no evidence of developmental toxicity in the F_1_ generation. Assessments showed no adverse effects on fetal body weight and morphology, postnatal growth and development, behavior, reproductive performance, and survival. Fetal morphological examinations indicated no negative effects in the BPV- and BAA-treated groups compared to the PBS control group. Additionally, examinations of maternal thoracic and abdominal organs—such as the brain, heart, lungs, thymus, kidneys, and spleen—showed no adverse impacts on the groups treated with BPV and BAA compared to the PBS control group. Furthermore, hematological and biochemical parameters for both mothers and offspring remained unchanged in the BPV group compared to rats in the PBS control group on day 90.

### 3.2. Assessment of Local Tolerance in Rats

Intramuscular irritation studies in rats revealed mild-to-moderate inflammation and redness at injection sites in the BAA- and vaccine-treated groups. Histomorphological assessments indicated no substantial tissue damage. While local reactions persisted, signs of recovery were evident by day 90.

### 3.3. Cell-Mediated and Humoral Immune Responses in BALB/c Mice After Immunization

#### 3.3.1. Lymphocyte Proliferation and Production of IFN-γ and IL-4 After Three Immunizations in BALB/c Mice

In this study, we compared lymphocyte proliferation following in vitro BPV stimulation among PBS control, BAA-treated, and vaccine-treated groups, based on the methodology of Linda Mull et al. [17]. To address concerns regarding potential overstimulation of immune system by the vaccine, lymphocytes were restimulated in vitro with BPV. A major limitation of this study was the use of the whole vaccine rather than purified antigen for lymphocyte stimulation. Spleen cells were collected on days 14 and 70—two weeks after the final injection—to evaluate cell proliferation and cytokine production. Splenocytes stimulated with 15 µg/mL of BPV showed proliferation in mice treated with BPV, PHA, and cell culture media on day 70 (Figure 1a–d). Specifically, 26.5%, 56.39%, and 15.2% of the lymphocytes proliferated in response to BPV, PHA, and cell culture media, respectively. The pattern and percentage of proliferated lymphocytes in BPV-immunized mice were comparable to those in the GV group (*p* < 0.05). The results were based on cells obtained from immunized mice, which were divided into four groups: those that received the vaccine (BPV and GV), BAA treatment, and PBS control. All groups stimulated in vitro using the BPV. Splenocytes from mice treated with BPV or GV showed significantly higher proliferation than those from PBS-treated mice. The data demonstrated increased CD3^+^, CD4^+^, and CD8^+^ T-cell proliferation in vaccine-treated groups compared to PBS controls. Compared to the negative (cell culture only) and positive (PHA-treated) controls, PBS-treated mice exhibited increased splenocyte proliferation following in vitro stimulation, although the response was less pronounced than that observed in vaccine-treated groups. The BAA-treated group showed a weaker proliferative response than the GV and BPV groups, suggesting that the antigens in BPV are necessary for initiating specific T-cell responses.

Isolated splenocytes from mice were stained with FITC anti-mouse CD3, PE-conjugated anti-mouse CD4^+^, and APC-conjugated anti-mouse CD8a antibodies. On day 14 post-vaccination, no increase in the percentage of CD3^+^ lymphocyte was observed in mice from groups A_1_ (BPV), B_1_ (GV), C_1_ (PBS, negative control), and D_1_ (BAA) following exposure to PHA, BPV, or cell culture media (Figure 2a). However, a significant increase in CD3^+^ lymphocytes was noted in BPV- and GV-immunized mice on day 70, as compared to day 14 (Figure 2a). As shown in Figure 1e–f and Figure 3a, there was no significant difference in the percentage of CD3^+^ lymphocytes between the BPV and the GV groups on day 70. However, statistically significant differences were observed when comparing BPV with BAA and PBS groups (*p* < 0.01 and *p* < 0.001, respectively). Compared to PBS-treated mice, both BPV- and GV-immunized groups showed a significant increase in CD3^+^ lymphocyte percentages on day 70 (Figure 2a). Flow cytometry analysis revealed that the CD4^+^ lymphocyte populations increased by approximately 20% in BPV-treated group and 40% in GV-treated group compared to other groups on day 70 (Figure 1g–j). The increase in CD4^+^ lymphocytes in the BPV (A_1_) and GV (B_1_) groups was comparable and not statistically different (Figure 1g,h and Figure 2b). On day 14, no change in CD4^+^ lymphocyte levels were observed in vaccine-treated (BPV and GV) or BAA-treated groups compared to PBS controls (Figure 2b). However, CD4^+^ lymphocyte counts significantly increased from day 14 to day 70 in BPV- and GV-treated groups, with BPV showing a significant increase compared to the BAA (*p* < 0.05) and PBS (*p* < 0.01) groups (Figure 2b). No significant increase in CD8^+^ lymphocyte percentages was observed on day 14 in vaccine- or BAA-treated groups compared to PBS controls (*p* > 0.05). However, a significant increase was noted on day 70 (*p* < 0.05), with CD8^+^ populations rising by approximately 8% in BPV- and GV-treated groups compared to PBS controls (Figure 1k–n and Figure 2c). Cytotoxic lymphocyte counts increased significantly from day 14 to day 70 in BPV- and GV-treated mice (*p* < 0.001), with no significant difference between BPV and GV groups on either day (*p* > 0.05; Figure 1k,l and Figure 2c). As shown in Figure 2c, CD8^+^ lymphocyte percentages in BPV-immunized mice were significantly higher than those in BAA- and PBS-treated groups (*p* < 0.01 and *p* < 0.001, respectively). In test wells from BPV-, GV-, and BAA-treated groups, IFN-γ levels were elevated on days 14 and 70 post-injection compared to the PBS controls, with a statistically significant increase observed on day 70 (*p* < 0.0001 for BPV and GV and *p* < 0.001 for BAA; Figure 2d). No significant difference in IFN-γ induction was observed between BPV (A_1_) and GV (B_1_) groups on either day (*p* > 0.05; Figure 1g,h). However, IFN-γ levels significantly increased in all treated groups from day 14 to day 70 compared to PBS controls (Figure 2d). The difference in IFN-γ levels between BPV and BAA groups was significant (*p* < 0.001), and even more pronounced between BPV and PBS groups on day 70 (*p* < 0.0001). IL-4 levels significantly increased in the vaccine- and BAA-treated groups from day 14 to day 70 (*p* < 0.05; Figure 2e). No significant difference was observed in IL-4 levels between the vaccine-treated and the BAA-treated groups on either day (*p* > 0.05; Figure 2e). Positive control wells (PHA-stimulated splenocytes) in each of the four immunized mouse groups showed a significant rise in lymphocyte percentages compared to their respective negative controls on the same day.

#### 3.3.2. Serologic Analysis

**Figure 4:**
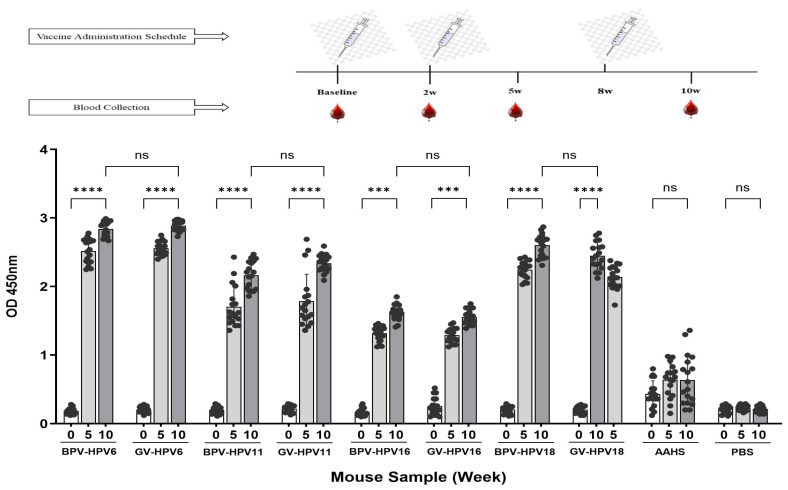
BPV vaccination induced IgG antibody responses against all four L1 subtypes (HPV6, 11, 16, and 18) in female BALB/c mice, as determined by ELISA. Individual antibody titers as well as group mean responses are shown. Humoral immune response data were analyzed using the non-parametric Kruskal–Wallis test. Each group consisted of five animals (n = 18): group 1 (BPV), group 2 (GV vaccine), group 3 (BAA), and group 4 (PBS). Measurements were obtained three weeks after the second immunization and two weeks after the third immunization. Antibody titers are presented as mean values with 95% confidence intervals (CIs). A *p* value < 0.05 was considered statistically significant. *** *p* < 0.001; **** *p* < 0.0001; ns, not significant.

Total IgG levels were assessed using an indirect ELISA test. A mouse anti-HPV L1 ELISA was employed to detect IgG antibodies specific to the HPV L1 protein from HPV types 6, 11, 16, or 18. The results demonstrated that immunization of mice with BPV or GV significantly increased specific IgG antibody responses compared to the group treated with BAA (*p* < 0.05). No significant difference was found between the BPV and the GV groups. This study confirmed that antibody titers persisted on day 70. Increases in antibody levels on days 35 and 70, compared to pre-immunization levels, were evaluated by comparing vaccine-treated groups with the PBS control group (Figure 4). All pre-vaccination samples showed antibody levels below the cut-off value of 1 U/mL.

## 4. Discussion

Several commercial vaccines targeting low-risk and high-risk HPV types have been developed to protect women from cervical cancer caused by HPV infection [11]. The newly developed BPV must be evaluated for immunogenicity and safety [17]. We developed a new quadrivalent HPV vaccine (BPV) containing adsorbed monovalent HPV L1 proteins from types 6, 11, 16, and 18. Specific antigenic content was used in each study (Table 1). Since there was no animal model for HPV infection to analyze vaccine protection [17], this study focused on evaluating the pharmacology, immunogenicity, and safety of BPV. This study aimed to assess immunogenicity and local and systemic toxicity in male and female BALB/c mice and reproductive toxicity in female Wistar rats, followed by a recovery period. The study revealed that (i) no changes in hematological, biochemical, gross pathology, and histopathology parameters were associated with BPV during the observation period, (ii) local reactions at BPV injection sites in rats resolved after 90 days, (iii) in vitro BPV stimulated lymphocyte proliferation, (iv) BPV exposure led to the expansion of CD3^+^, CD4^+^, and CD8^+^ T lymphocytes, (v) BPV increased secretion of IFN-γ and IL-4 in cell cultures, and (vi) BPV induced the production of anti-HPV L1 antibodies (types 6, 11, 16, and 18) following a three-dose regimen.

Immunogenicity results for BPV were comparable to those of GV, which served as the positive control. A sub-chronic toxicity study in mice revealed minor but statistically significant changes in blood parameters (ALT, AST, ALP, GLU, BUN, Crea, urea, RBC, Hgb, Hct, MCV, MCH, MCHC, PLT, and neutrophils) compared to controls, all of which normalized by day 90 (*p* < 0.05).

Gross pathology findings related to BPV and BAA included increased splenic weight at final necropsy, which returned to normal by day 90. The PBS-treated group exhibited increased liver weight, suggesting this is unrelated to antigenic histomorphological changes observed in the BPV-treated group. The BAA-treated group showed a minor decrease in lymphoid tissue in the white pulp of spleens, swelling of kidney tubular epithelial cells, granulocyte infiltration, necrotic skeletal muscle fibers at the injection site, and a mild influx of inflammatory cells in the liver tissue. By day 60, the slight lymphoid depletion in the white pulp of the spleen and the swelling of renal epithelial cells had resolved. By day 90, inflammation at the injection site and mild infiltration of inflammatory cells in the liver parenchyma had improved. While histopathological changes in both BPV-treated and BAA-treated groups were notable, all adverse effects resolved, and the administered dose was deemed suitable for vaccine testing in mice. All reported adverse events in the spleen, injection site, and kidney at high doses were within normal limits at recovery necropsy. Human clinical trials of the quadrivalent HPV vaccine (BPV) have also been completed, and data will be published soon. Similar adverse events and recovery patterns were reported by David Wise et al. [26].

The repeated administration of BPV and BAA to rats did not result in maternal toxicity, including effects on fertility metrics, mating outcomes, and gross anatomical abnormalities of the thoracic and abdominal organs (the brain, heart, lungs, thymus, kidneys, and spleen) as observed on day 90. There was also no evidence of fetal toxicity, including parameters related to fetal survival, weight, morphology, physical signs, and growth. Additionally, hematological and biochemical parameters in both the mothers and F1 generation did not differ between BPV-immunized rats and those injected with PBS on day 90. These findings indicate that BPV did not produce adverse effects on reproductive health.

Reproductive toxicity findings of our study were consistent with those reported for Cervarix and Gardasil [19,27,28]. Similarities in local redness, hardness, and inflammatory response were noted at injection sites in both BPV and GV groups. Lymphocyte proliferation rates were recorded at 26.5% for BPV, 56.3% for PHA, and 15.2% for cell culture media (Figure 1a–d). These results indicate a significant increase in lymphocyte populations upon re-exposure to BPV in previously immunized mice. T-lymphocyte proliferation, a key feature of adaptive immunity, indicates the potential of immune system to react with exogenous antigens [24]. Significant increases in CD3^+^, CD4^+^, and CD8^+^ T lymphocytes were observed in BPV-treated mice on day 70 compared to PBS-treated controls (Figure 1e–n and Figure 2a–c), which is consistent with previous studies [21]. The observed increase is likely due to the prior antigen exposure and the development of immune memory. Elevated markers in splenocytes of BAA-treated mice were presumably linked to the immunostimulatory and immunopotentiation effects of the adjuvant [29]. Given the vital role of T cells in producing adaptive antibody responses, controlling the progression of immune responses, forming immunological memory, functioning effectively against infected and abnormal cells, and ultimately clearing pathogens, our study observed the substantial proliferation of the lymphocyte population induced by BPV [22].

In this study, a significant increase in Th1 (IFN-γ) and Th2 (IL4) levels was observed in the splenocyte culture supernatant of mice immunized with BPV compared to those that received PBS on day 70. Given the roles of IFN-γ in antiviral activity, immune regulation, and anti-replicative effects on HPV, the rise in this cytokine following BPV exposure was particularly noteworthy. IL-4 levels were also elevated in both the BAA- and vaccine-treated groups compared to the PBS control group on day 70 (Figure 1i,j). These findings demonstrate that immunizing BALB/c mice with BPV induces a potentially protective T-cell response through the production of both Th1 and Th2 cytokines [26]. Vaccine-treated groups also exhibited high levels of serum IgG antibodies against HPV VLPs for types 6, 11, 16, and 18 compared to the PBS control group. The increase in antibody titers on day 70 compared to day 35 indicates the booster effect of the third dose. However, since blood sampling on day 70 was performed only 14 days after the third immunization, the antibody titers may not have reached their peak level yet. Notably, comparable antibody titers were observed between the BPV- and GV-vaccinated groups across all HPV types (6, 11, 16, and 18), suggesting that the candidate BPV elicits a humoral immune response similar in magnitude and kinetics to that of the commercial quadrivalent vaccine. This comparable performance highlights the strong immunogenicity of BPV and supports it as an HPV vaccine candidate. These findings are consistent with previous studies on HPV vaccines in BALB/c mice. It should be acknowledged that a limitation of our study is the reliance on anti-L1 IgG ELISA as the only immunogenicity assessment. Although ELISA-detected binding antibodies are not identical to neutralizing antibodies, prior research has consistently demonstrated an association between anti-L1 antibody levels and protective immune outcomes [30]. Accordingly, our results primarily capture vaccine-induced binding responses, offering valuable insight into immunogenicity. We also emphasize that further studies incorporating PBNA or validated functional surrogates would be valuable to confirm the protective efficacy of the new designed vaccine. Due to observed structural instability of VLPs at room temperature and at 2–8 °C in our laboratory, VLPs were immediately loaded onto the BAA and used for in vitro stimulation, supporting findings reported by Pasupuleti et al. and Yasaghi et al. [31,32]. However, in vitro re-stimulation using the whole vaccine could simulate in vivo conditions but is not specific to our antigens owing to the presence of BAA and expedients. This issue was the main limitation of our study [33,34]. Although, the primary aim of this study was to evaluate the overall immunogenicity of the L1-VLP-based HPV vaccine rather than to define precise antigen-specific T-cell responses. Prophylactic HPV vaccines are inherently VLP-based and non-replicating, and their main protective mechanism is mediated through antibodies induced by L1-VLPs, while cellular data are generally reported as supportive findings [11,35].

To attribute lymphocyte proliferation results to VLP antigens and clarify the immune response, additional targeted experiments, such as cytokine analysis and flow cytometry, were performed, along with a benchmark control group (GV) [36]. Flow cytometry analysis revealed a significant rise in the proliferation of CD3^+^, CD4^+^, and CD8^+^ lymphocytes in the BPV and GV groups compared to the BAA group (Figure 2a–c). Additionally, our findings showed increased IL-4 secretion in both the BAA and BPV groups and a marked increase in IFN-γ levels in the BPV group (Figure 2d). Comparable results were also observed in the GV group. Since splenocytes from all four groups (BPV, GV, BAA, and PBS) were stimulated in vitro with the complete BPV formulation, and both BPV and GV groups exhibited significantly higher CD marker percentage and cytokine production compared to the BAA-only group, the observed effects may be indirectly linked to the antigen. Previous studies by Serre et al. and Li et al. have demonstrated that aluminum-based adjuvants predominantly drive the production of IL-4 and other Th2-associated cytokines, while exerting comparatively weaker effects on IFN-γ secretion. This pattern reflects the well-established tendency of aluminum salts to bias T-cell responses toward a Th2 phenotype, thereby favoring humoral immunity and antibody generation, but contributing minimally to cytotoxic T-cell activity. In this context, the increase in CD8^+^ T cells observed in our study is unlikely to be attributable to the BAA itself. Instead, it more plausibly reflects stimulation by the virus-like particles (VLPs), which are known to provide structural and antigenic cues capable of eliciting broader cellular responses. This distinction is important, as it underscores the complementary roles of adjuvants and VLP antigens: while aluminum enhances antibody-mediated protection, the VLP component may contribute to limited but measurable cellular activation. Future studies incorporating adjuvant-only and antigen-only control groups would be valuable to further disentangle these effects, allowing the clearer attribution of cytokine and proliferation outcomes to either innate adjuvant activity or antigen-specific adaptive immunity. Such an approach would strengthen mechanistic insights and align with the prior literature examining the independent and synergistic contributions of aluminum adjuvants and VLP antigens in shaping vaccine immunogenicity [36,37,38,39]. It is worth noting that the formulation and components of BPV, including the antigen, adjuvant, excipients, and their respective quantities, are designed to be consistent with those of Gardasil. In addition, BPV has undergone stability testing, demonstrating acceptable stability for up to two years. While these findings support formulation comparability, further long-term studies are required to fully assess potential differences in immune persistence and long-term safety. As vaccine stability was not considered in the study design, this aspect has not been included in this article.

## 5. Conclusions

This study provides comprehensive evidence that the administration of BPV to both mice and rats was well tolerated and did not elicit any detectable toxicological effects. The careful monitoring of multiple biological parameters—including body weight, organ weight, histopathological examinations, and biochemical and hematological indices—revealed no deviations from normal physiological ranges. Importantly, reproductive assessments demonstrated that maternal animals and their F1 offspring were unaffected, thereby excluding concerns regarding potential generational toxicity. Beyond the absence of adverse effects, BPV exhibited clear immunological benefits. The compound successfully enhanced humoral immune responses, as evidenced by increased antibody production, and simultaneously promoted cellular immunity through the activation of T-cell responses. The balanced induction of Th1 and Th2 cytokines highlights BPV’s ability to orchestrate a protective and well-regulated immune profile, which is critical for effective prophylaxis and therapeutic applications.

Taken together, these findings underscore both the safety and immunogenic potential of BPV. The absence of toxicological concerns, coupled with its capacity to stimulate robust immune system, supports the advancement of BPV into human clinical trials. These results lay a solid foundation for future translational research, where BPV may contribute to the development of safe and effective interventions for human health.

## Figures and Tables

**Table 2:** Comparison of hematological and biochemical parameters in BALB/c mice were injected with four groups on day 90.

Hematological and Biochemical Parameters	Content of Injection Groups (Mean ± SD)			
	Adjuvant	BP	GV	PBS
RBC (10^9^/mcL)	6.8 ± 0.141	8.4 ± 0.1	7.533 ± 0.351	7.5 ± 0.35
Hb (g/dL)	15 ± 1.697	16 ± 1.2	13 ± 0.529	12 ± 0.5
Hct (%)	50.5 ± 0.707	42.5 ± 0.5	39.3 ± 1.587	38.7 ± 1.5
MCV (fl)	60 ± 0.0	58 ± 0.00	45.23 ± 11.46	51.4 ± 10.15
MCH (pg/cell)	22.5 ± 0.707	19.5 ± 0.5	17.233 ± 0.252	20.2 ± 0.25
MCHC (g/dL)	38.5 ± 0.707	36.5 ± 0.5	35.667 ± 0.577	34 ± 0.5
PLT (10^9^/L)	659 ± 58.196	570 ± 139	552.667 ± 96.194	491 ± 5.187
WBC (10^9^/L)	18.55 ± 2.192	17.55 ± 1.55	16.25 ± 1.061	8.25 ± 0.75
Neutrophiles	34 ± 11.747	32.778 ± 10.721	30 ± 9.101	25.4 ± 8.792
Eosinophiles	1.2 ± 1.211	1	1	0
Lymphocyte	64.6 ± 13.297	66 ± 12.359	69.01 ± 8.5	74.167 ± 9.928
Monocyte	0.6 ± 0.894	1.111 ± 1.691	0.857 ± 1.167	1 ± 0.632
ALT (IU/L)	220 ± 26.87	189 ± 19	175.667 ± 8.021	140 ± 7.041
AST (IU/L)	165.5 ± 31.82	152.5 ± 22.5	130.333 ± 3.512	110 ± 3.5
ALP (IU/L)	95.5 ± 21.190	80.5 ± 5.134	96 ± 13.231	80 ± 5.218
Urea (mg/dL)	18.5 ± 9.192	19.5 ± 6.5	16.25 ± 4.856	17 ± 5.75
Crea (mg/dL)	1.3 ± 0.354	0.9 ± 0.25	1.1 ± 0.096	1.2 ± 0.125
Animals per groups (n)	6	6	6	6

RBC: red blood cell; Hb: hemoglobin; Hct: hematocrit; MCV: mean corpuscular volume; MCH: mean corpuscular hemoglobin; MCHC: mean corpuscular hemoglobin concentration; PLT: platelet; WBC: white blood cell; ALT: alanine aminotransferase; AST: aspartate aminotransferase; ALP: alanine aminotransferase; Crea: creatinine.

## Data Availability

The original contributions presented in this study are included in the article. Further inquiries can be directed to the corresponding author.
